# CENTER FOR SCIENTIFIC REVIEW: YOUR APPLICATION’S JOURNEY BEGINS WITH THE DIVISION OF RECEIPT AND REFERRAL

**DOI:** 10.1093/geroni/igae098.1802

**Published:** 2024-12-31

**Authors:** Marc Boulay

**Affiliations:** National Institutes of Health, Baltimore, Maryland, United States

## Abstract

The first stop for your application is at the Division of Receipt and Referral (DRR), the central receiving point for all competing grant applications submitted to the NIH. Here, your application will receive: (a) an initial check for its compliance with both NIH policy and the specific requirements of the Notice of Funding Opportunity used for the submission, (b) an assignment to a funding Institute, and (c) an assignment to the most appropriate study section for review. Attendees will become acquainted with the process used by DRR and will receive some tips to help them avoid common errors with their submission. This session will also highlight tools that applicants may use to inform their application’s review assignments and will be introduced to the ENQUIRE process (Evaluating Panel Quality in Review), ongoing evaluations of CSR’s study sections to ensure that the scientific scope of the study sections align with the current state of the science. By the end of the presentation, attendees should have a greater understanding of the factors they should consider when preparing their NIH submissions.

